# Consistent DNA Hypomethylations in Prostate Cancer

**DOI:** 10.3390/ijms24010386

**Published:** 2022-12-26

**Authors:** Marcos J. Araúzo-Bravo, Lars Erichsen, Pauline Ott, Agnes Beermann, Jamal Sheikh, Daniela Gerovska, Chantelle Thimm, Marcelo L. Bendhack, Simeon Santourlidis

**Affiliations:** 1 Computational Biology and Systems Biomedicine, Biodonostia Health Research Institute, 20014 San Sebastián, Spain; 2 IKERBASQUE, Basque Foundation for Science, 48009 Bilbao, Spain; 3 Department of Cell Biology and Histology, Faculty of Medicine and Nursing, University of Basque Country (UPV/EHU), 48940 Leioa, Spain; 4 Epigenetics Core Laboratory, Medical Faculty, Institute of Transplantation Diagnostics and Cell Therapeutics, Heinrich Heine University Düsseldorf, 40225 Düsseldorf, Germany; 5 Medical Faculty, Institute for Stem Cell Research and Regenerative Medicine, Heinrich Heine University Düsseldorf, 40225 Düsseldorf, Germany; 6 Department of Urology, University Hospital, Positivo University, Curitiba 80420-011, Brazil

**Keywords:** epigenetics, hypomethylation, diagnosis, prostate cancer, democratic method

## Abstract

With approximately 1.4 million men annually diagnosed with prostate cancer (PCa) worldwide, PCa remains a dreaded threat to life and source of devastating morbidity. In recent decades, a significant decrease in age-specific PCa mortality has been achieved by increasing prostate-specific antigen (PSA) screening and improving treatments. Nevertheless, upcoming, augmented recommendations against PSA screening underline an escalating disproportion between the benefit and harm of current diagnosis/prognosis and application of radical treatment standards. Undoubtedly, new potent diagnostic and prognostic tools are urgently needed to alleviate this tensed situation. They should allow a more reliable early assessment of the upcoming threat, in order to enable applying timely adjusted and personalized therapy and monitoring. Here, we present a basic study on an epigenetic screening approach by Methylated DNA Immunoprecipitation (MeDIP). We identified genes associated with hypomethylated CpG islands in three PCa sample cohorts. By adjusting our computational biology analyses to focus on single CpG-enriched 60-nucleotide-long DNA probes, we revealed numerous consistently differential methylated DNA segments in PCa. They were associated among other genes with *NOTCH3, CDK2AP1, KLK4,* and *ADAM15*. These can be used for early discrimination, and might contribute to a new epigenetic tumor classification system of PCa. Our analysis shows that we can dissect short, differential methylated CpG-rich DNA fragments and combinations of them that are consistently present in all tumors. We name them tumor cell-specific differential methylated CpG dinucleotide signatures (TUMS).

## 1. Introduction

Prostate cancer (PCa) is a devastating disease significantly threatening the health of every single man, mainly in the last third of his lifespan. E.g., in the United States, the lifetime risk of being diagnosed with PCa is approximately 13%, and the lifetime risk of dying of PCa is 2.5% [1]. The estimated new cases and deaths in 2020 for the whole world were 1,414,259 and 375,304, respectively [2].

For many decades, the currently applied PCa diagnosis standards and radical treatment methods have basically remained unchanged in clinical practice. Nevertheless, they are not optimized and satisfyingly balanced in respect of benefit and harm, as recognized not only by any expert in the field, but also by patients with the appropriate knowledge. This is underlined by the fact that recommendations against prostate-specific antigen (PSA) screening, e.g., the 2012/2018 US Preventive Services Task Force Guidelines, were made despite the evidence that screening reduced the risk of developing metastatic PCa and dying from the disease [3,4].

These recommendations resulted i.a. from the adverse effects of PSA screening for many men with a slow-growing, indolent PCa that otherwise would have never been noticed during the men’s lifetimes. Overdiagnosis and overtreatment turn those men into patients, burden them with psychological stress and a deteriorating quality of life, and if surgery and radiation is applied, a substantial proportion of those men face urinary (incontinence), sexual, and bowel dysfunction, and in some cases even for many years after treatment [3].

Therefore, it is of high priority to supplement the actual PSA testing technology with more sensitive and robust PCa biomarkers in order to reduce overtreatment, and additionally consider more often active surveillance [4] and preserving therapies, e.g., high-intensity focal ultrasound (HIFU).

Current DNA methylation biomarkers have the potential to relieve this situation [5,6]. DNA methylation occurs at CpG dinucleotides interspersed throughout the whole genome, many of them organized in CpG dense clusters, named CpG islands, associated with 5′ regulatory regions of approximately 60% of the genes, and are also located in inter and intragenic CpG-rich regions of known or unknown regulatory function [7]. Gene promoter methylation is associated with chromatin condensation and gene silencing, whereas gene promoter hypomethylation correlates with accessibility for transcription initiation [7,8]. 

Furthermore, it has been shown that differential methylation at individual CpG dinucleotides affects gene expression [9]. Such differential methylated CpG signatures reflect the cellular composition of cellular mixtures and different tissues [9], and have been implicated in cancer prognosis [10]. Therefore, they have been suggested to be promising for stratification of cancer samples [9].

Since aberrant DNA methylation associated with tumorsuppressor and oncogenes is involved in the initiation and progression of carcinogenesis [11], several studies have already investigated CpG islands DNA methylation in PCa [12,13,14]. Hence, novel differential methylated genes have been reported which correlate with PCa and high-grade prostatic intraepithelial neoplasias, or have potential functional consequences in PCa or the potential to distinguish PCa from adjacent benign tissue [15,16,17,18]. These studies provide clear evidence for the importance of aberrant DNA methylation for PCa diagnosis.

However, to use cancer cell-associated differential methylation for diagnostic and prognostic purposes, a distinct differential methylated region (DMR) has to consistently prevail in an early tumor stage and in a distinct class of tumors with a specific clinical behavior. This is the fundament of a potent biomarker to achieve high sensitivities and specificities in clinical application, firstly for early diagnosis and secondly for a substantiated prognosis.

DNA hypomethylation is a ubiquitous feature of carcinogenesis affecting various cancers, including PCa [19]. It is often observed during the early stages of tumorigenesis, and generally more pronounced with tumor progression or the degree of malignancy [19]. Hypomethylation events have been described to be involved in reactivation of oncogenes, genes associated with tumor invasion or metastasis [20]. Furthermore, hypomethylation comprises genome-wide distributed retrotransposons, e.g., LINE-1, which are thought to play one causative role in cancer [21]. LINE-1 hypomethylation increases with tumor grade and stage, and is particularly pronounced in lymph node-positive prostate tumors [22]. It has been suggested that in the future, DNA hypomethylation markers will be examined for their methylation status to characterize cancers and design the best treatment for a given tumor and individual [19].

Our findings here reveal many new genes being associated with differential methylated CpG islands in PCa. Additionally, we found many new distinct hypomethylated 60 bp DNA segments which exhibit a higher degree of consistent prevalence in PCa samples in comparison to hypomethylated CpG islands. Thus, these findings show the promise and importance of short CpG-rich DNA segments in the development of a dynamic epigenetic biomarker.

## 2. Results

At a starting point of our study, some principal questions came up: can we detect useful differential methylations in tissue samples from PCa patients at all? Naturally, this tissue material is of a rather heterogenic cellular composition. What would the methylation results look like when, e.g., mixed samples have to be analyzed? Would the differential methylations be more informative from biopsies or from primary cancer tissue samples?

### Distinct Hypomethylated 5′ Gene Regions in Tissue Specimens of PCa Patients 

We started to produce genome-wide DNA methylation data sets by MeDIP and by gene promoter array analyses from pathologically reviewed and classified prostate gland tissue samples from biopsies and primary tumors (clinical center No. 1, see M&M). The samples were chosen from five patient biopsies of Gleason 7 (3 + 4) and five patient primary tumor samples of Gleason 7 (4 + 3). These were two different patient cohorts. In each of them we picked up five samples of mixed tissue, consisting of nearly 50% tumor-adjacent, normal-appearing gland tissue and 50% of tumor tissue, five samples of tumor-adjacent, healthy gland tissue, and five samples of tumor tissue consisting of >90% of cancer cells. The reference group, to which all other groups were compared, consisted of five samples, each with >90% of benign prostatic hyperplasia cells (BPH).

**Figure 1:**
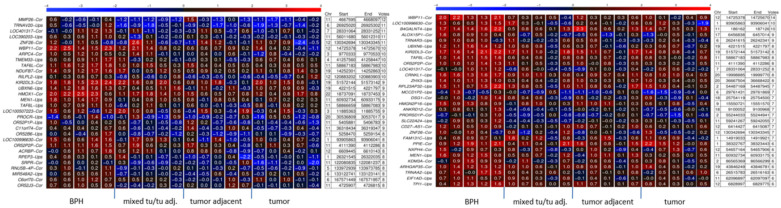
DNA methylation heat maps of CpG-rich 5′ gene regions from PCa biopsies (G7, 3 + 4) and PCa primary tissue samples (G7, 4 + 3) compared to BPH reference samples. Five tissue samples from BPH prostate glands, five mixed tissue samples of tumor and tumor-adjacent tissue (mixed tu/tu adj.; 50/50%), five samples of tumor-adjacent gland tissue (tumor adjacent) and five tumor tissue samples (tumor, >90%) were used. The left panel corresponds to biopsy samples and the right panel shows the results of primary PCa tissue samples. Only the first 30 most-voted differentially methylated loci out of 237 hypomethylated regions in the biopsies and 202 hypomethylated regions in the primary tumors are presented in each case. Each rectangle in the heat map stands for one CpG-rich 5′ gene region, with its chromosomal location indicated on the right-hand side (Chr, start/end). The “votes” number indicates how many of the samples were found with hypomethylation of the respective region by the democratic method. Redder color corresponds to more methylated regions. I.e., the most unmethylated 5′ gene regions are light blue. The grade of methylation is also indicated by numbers within the colored rectangles. Higher number corresponds to higher methylation. The abbreviation “Cor” after the gene name indicates an examined CpG-rich region of −500 to +500 nucleotides surrounding the transcription start site (TSS). A further examined region of −1500 to −500 bases lying upstream of the TSS is referred to as “Ups” following the gene name.

In total, we detected 237 hypomethylated 5′gene regions in the biopsies and 202 in the primary tumor samples, compared to the BPH reference group. Figure 1 presents a small excerpt of the 30 top DMRs selected by our computational democratic method for each case, the biopsies (left panel) and the primary tumor samples (right panel). The hypomethylated regions were distributed among all three analyzed subgroups in each case, where overall hypomethylation appears more pronounced in the biopsies, and with hypomethylations also being detected within the primary tumor samples. Interestingly, we detected several common differential methylated markers in these two different patient cohorts. Among them are, e.g., *WBP11*, *MEN1*, *OR52P2P*, *KIR2DL3, TAF6L, UBXN6* as shown in Figure 1, and a few others, e.g., *KDM3A* and *TMEM33*.

**Figure 2:**
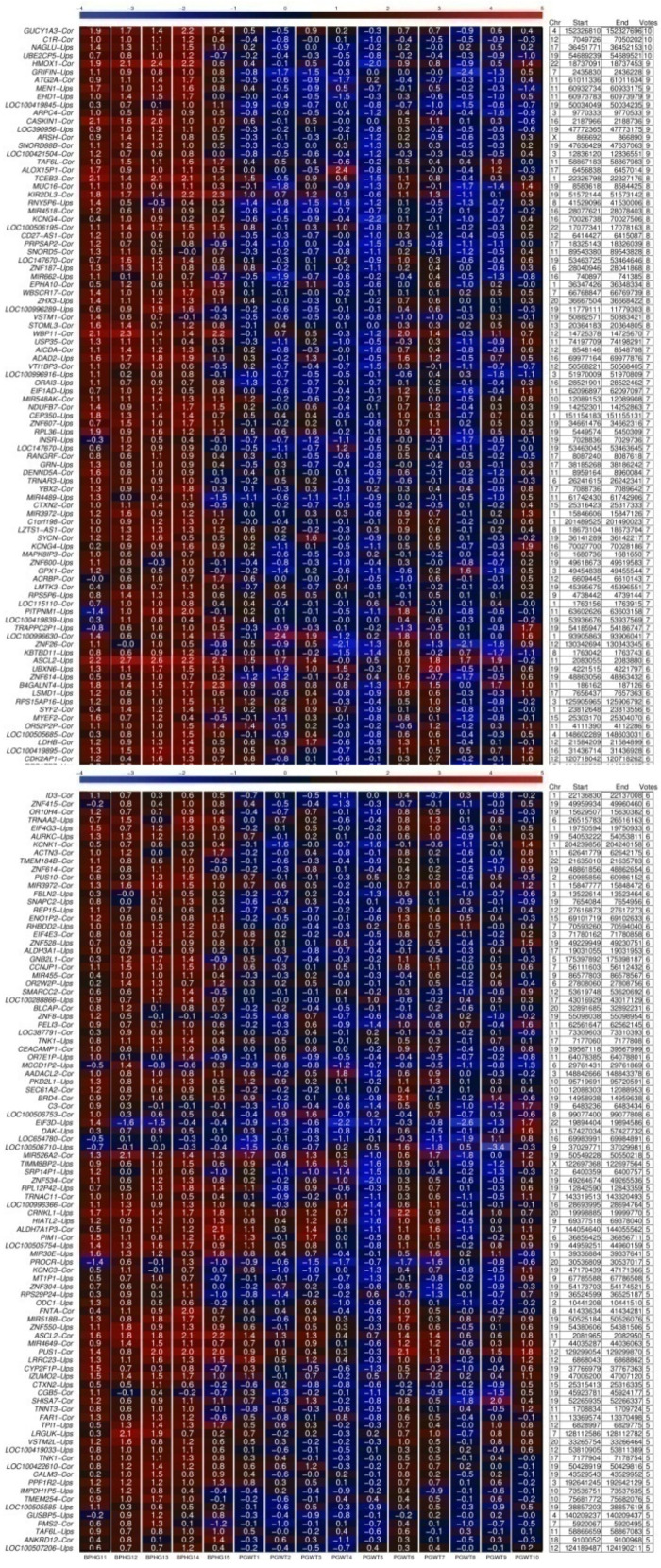
DNA methylation heat maps of CpG-rich 5′ gene regions in BPH samples and PCa tissue samples. A total of 5 tissue samples from BPH prostate glands and 10 PCa tumor tissue samples were used (tumor). The top 186 most voted by our democratic method differentially methylated loci out of 370 hypomethylated regions found are presented from left to right. Both panels correspond to results from the same primary PCa tissue samples of various tumor stages and grades provided by clinical center No. 2 (see M&M section). Each rectangle in the heat map stands for one CpG-rich 5′ gene region, with chromosomal location indicated on the right hand side (Chr, start/end). The “votes” number indicates how many out of the 10 PCa samples were found by the democratic method with hypomethylation of the respective region compared to the 5 BPH samples. Redder color corresponds to more methylated regions. E.g., the most unmethylated 5′ gene regions are light blue. The grade of methylation is also indicated by numbers within the rectangles in the heat maps. Higher number corresponds to higher methylation. The abbreviation “Cor” after the gene name indicates an examined CpG-rich region of −500 to +500 bases surrounding the transcription start site (TSS). A further examined region of −1500 to −500 bases lying upstream of the of TSS is referred to as “Ups” following the gene name.

Once we have confirmed that hypomethylation was detectable by this approach, we screened for hypomethylated gene regions also in prostate tumor samples from another independent clinical center in Germany. This center, No. 2, provided 10 pathologically reviewed and precisely excised prostate tumor samples (>95% cancer cells) of various tumor stages and grades (Gleason 7 (3 + 4) and (4 + 3), see M&M). In Figure 2 we show the most consistently hypomethylated 186 of in total 370 gene regions found. In this experiment, we detected distinct hypomethylation of more gene regions in the tumor samples compared to the reference ones. Additionally, the grade of consistency appears higher with several DMRs with 10, 9, and 8 votes, which means that they have been found to be significantly hypomethylated in 10, 9, and 8 out of 10 tumor samples in comparison to the average methylation degree of all five BPH reference samples. In Table 1 we summarize candidates which were found to be simultaneously differential hypomethylated in PCa vs. BPH in all three so far examined different patient cohorts. We refer to the function of these genes, and in each case we present one selected published study which provides evidence for functional involvement of the respective gene in PCa and cancer in general.

**Figure 3:**
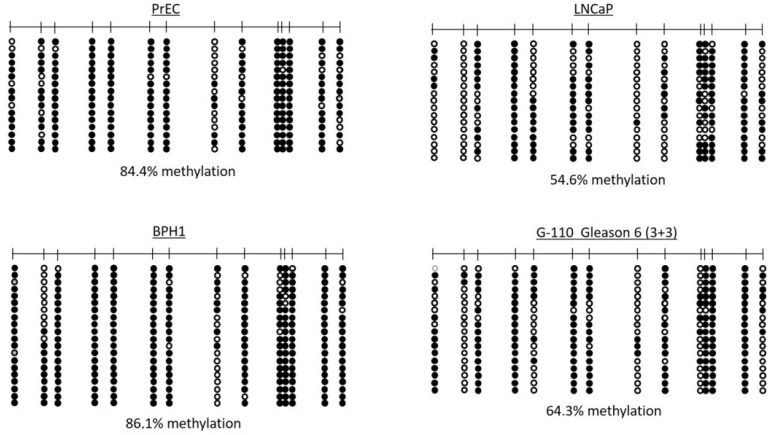
Lollipop diagrams of the bisulfite genomic sequencing analyses of the methylation status of each CpG of the *KIR2DL3* promoter in the prostatic epithelial cell line PrEC, the PCa cell line LNCaP, one BPH tissue sample of >95% BPH cells, and one PCa tissue sample of >95% cancer cells from a Gleason 6 tumor (3 + 3). Black, white, and grey circles stand for methylated, unmethylated, and undefined CpG dinucleotides, respectively.

For instance, among these genes we found *WBP11*, *B4GALNT4*, *KIR2DL3*, *TAF6L*, *MEN1*, *HMOX1*, *WBSCR17*, *NDUFB7*, *BRD4*, *PPFIA3*, *MIR4518*, *EPH10*, *ORAI3*, and *CDK2AP1*. All candidates except BRD4 show overexpression in “The Cancer Genome Atlas”, TCGA, PCa expression data bank [23,24]. Former data of ours obtained with the same MeDIP/array methodology had been previously verified with the technically independent method of bisulfite genomic sequencing [39,40]. Nevertheless, in our present study, we also chose the gene *KIR2DL3* to reaffirm the hypomethylation found by MeDIP. This gene is well described to be clonotypically expressed exclusively in small lymphocyte populations, primary NK cells, and only there it exhibits a hypomethylated gene promoter [41,42]. As known so far, it is supposed to be densely methylated in all other cells of our body, and therefore, if hypomethylated in PCa, it appears of predominant interest for diagnosis. In Figure 3 the respective small *KIR2DL3* CpG island was found to encompass 84% and 55% of methylation in the epithelial prostatic cell line PrEC and the PCa cell line LNCaP, and 86% and 64% of methylation in one BPH and one of our PCa tissue samples (pT2b G6, 3 + 3). Of note, such subsequent analyses revealing the most hypomethylation sensitive CpG dinucleotide positions in detail may later be used to identify the most discriminating primer binding positions of other DMRs in order to establish sensitive, fast, and cost-effective methylation-specific PCR (MSPCR) assays.

Next, we performed a blinded internal validation by repeating MeDIP and array procedure for the first five PCa samples from primary tumor tissues, consisting of >90% of cancer cells, in parallel with the ten samples from clinical center No. 2 (>95% of cancer cells), and five BPH samples. Then, unblinding took place in the attendance of a national expert from urology, the director of the coordination center for clinical studies of the Heinrich Heine University, and a colleague. The result was that by comparison of the differential methylated CpG islands of the new data set to those of the first data sets, our computational biology method was able to identify correctly 14 out of 15 PCa, and four out of five BPH samples on the basis of the redetected same CpG island hypomethylations. In one case, the method misstated one tumor sample as a BPH sample. However, this tumor sample groups with the BPH samples on the basis of all methylated CpG islands hierarchical clustering, as shown in Figure S1. Thus, this internal validation confirmed methodical reproducibility within the same sample cohorts.

**Figure 4:**
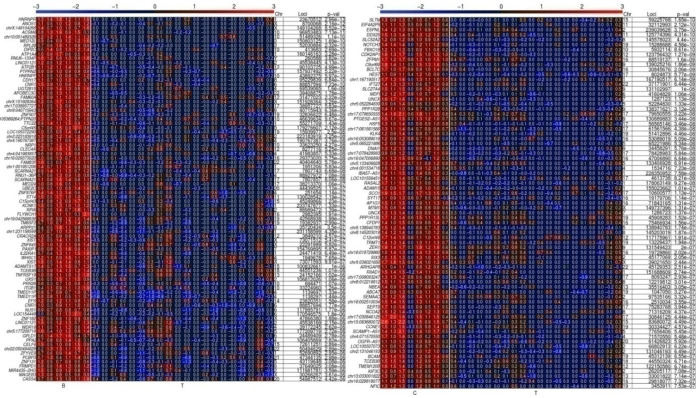
DNA methylation heat maps of CpG-rich DNA probes of 60 nucleotides associated with 5′ gene regions in BPH, PCa-adjacent and PCa tissue samples. Both groups, firstly, 6 tissue samples from BPH prostate glands (B), and secondly, 8 samples of tumor-adjacent healthy-appearing gland tissue (C), were separately analyzed in comparison to the same 20 PCa tumor tissue samples (T). Only the first 75–85 most differentially methylated CpG-rich probes out of approx. 250 hypomethylated genetic regions found in each group are presented. Each rectangle in the heat map stands for one CpG-enriched 60-nucleotides-long probe associated with the named 5′ gene region, with the chromosomal location indicated on the right hand side (Chr, start/end). Redder colors correspond to more methylated regions. E.g., the most unmethylated 5′ gene regions are light blue. The grade of methylation is also indicated by numbers within the colored rectangles. A higher number corresponds to higher methylation.

**Figure 5:**
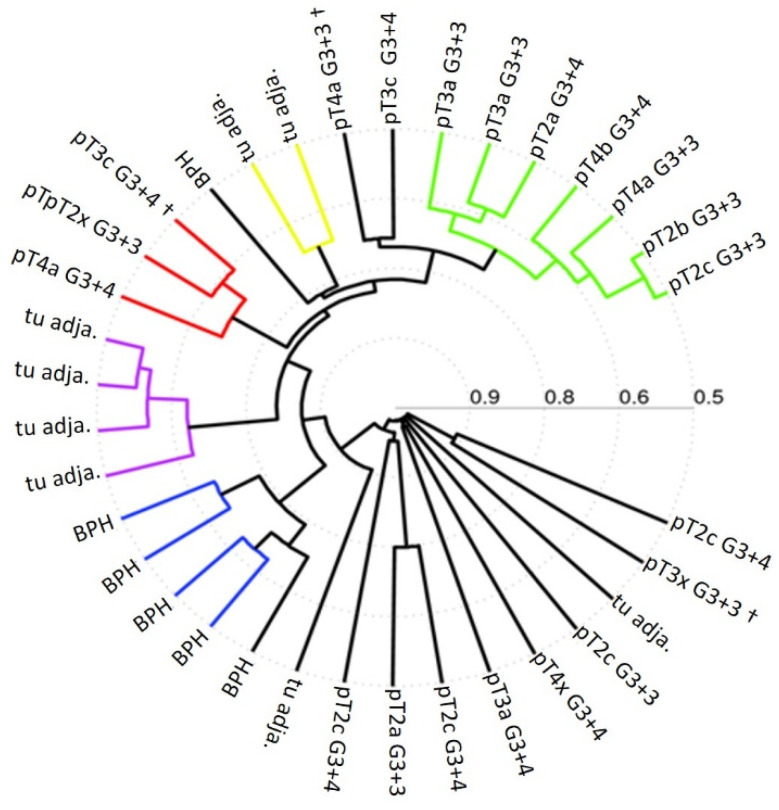
Hierarchical clustering of 6 tissue samples from BPH prostate glands, 8 samples of tumor-adjacent gland tissue (tu adja.), and 20 PCa tumor tissue samples (pT). It is performed using the correlation metric and the average linkage method. Illustrates relationship and group building based on distinct common DNA methylation signatures out of 2.0 million CpG-rich 60-nucleotide probes distributed over all CpG islands of the whole genome.

**Figure 6:**
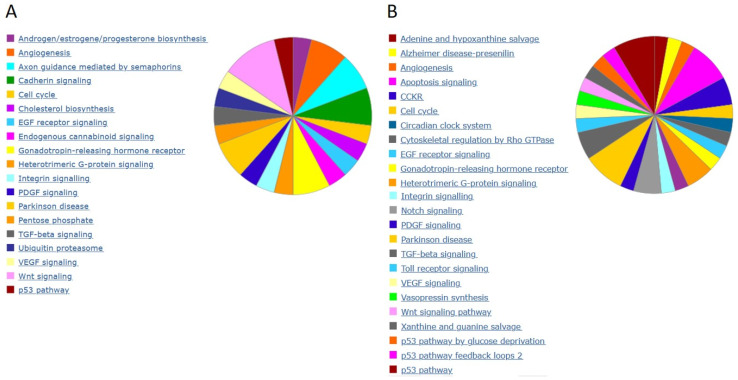
Panther analyses [38,43] of all depicted hypomethylated CpG probes in PCa samples vs. BPH (**A**) and vs. PCa adjacent healthy tissue (**B**) of Figure 4 reveal the involvement of the indicated pathways.

Although these results reveal some interesting common biomarker candidates, only few appear to consistently prevail in a hypomethylated state in all tumor samples. Therefore, we decided to slightly modify our approach in order to identify more hypomethylated genetic segments which would be consistently altered in all tumor samples. We decided to analyze new PCa samples and reference samples from a third independent clinical center. This clinical center, No. 3, provided 20 pathologically reviewed and classified prostate gland tissue DNA samples from primary tumors and 8 tumor-adjacent, healthy-appearing gland tissue samples as reference. In this experiment we adapted our computational biology analyses to focus on single CpG-enriched 60-nucleotide-long DNA probes, covalently bound at the spots of interest on the DNA methylation arrays. We detected 252 and 223 hypomethylated DNA probes in the tumor samples compared to BPH samples and tumor-adjacent, healthy-appearing gland tissue samples, respectively. In Figure 4 we show the heatmap of approx. 75 differential methylated probe loci for each case, compared to BPH (left panel) and to tumor-adjacent, healthy-appearing gland tissue samples (right panel). This result reveals numerous differentially methylated probes which are consistently hypomethylated in all 20 tumor samples compared to the reference samples. Furthermore, to explore the epigenetic relationship of all samples based on all the probes, we performed a hierarchical clustering which reveals an epigenetic classification of mainly three PCa sample groups (Figure 5). A blinded external validation was performed to confirm reproducibility by comparing the results of this external sample cohort from clinical center No. 3 with those of the previous analyses. We anonymously provided the array data from these prostate gland tissue DNA samples (clinical center No. 3) to the Coordination Center of clinical studies (KKS) Düsseldorf, where they compared blindly the DMRs based on 60bp probes with those based on whole gene promoter-associated CpG islands from the PCa samples provided by clinical center No. 2. A total of 40 differential methylated genes out of 100 were found again. Thus, these candidates have been externally validated. Panther analyses [43,44] for the evolutionary and functional classification of protein-coding genes based on a library of over 15,000 phylogenetic trees, performed with all detected, gene-associated, and hypomethylated CpG probes, reveal their association with PCa-involved genes and pathways, e.g., angiogenesis, apoptosis, cell cycle, NOTCH and WNT signaling, and p53 pathway (Figure 6). 

Finally, we summarized in Table 2 selected gene candidates which have been found to harbor the found hypomethylated probes from the tumor vs. tumor-adjacent experiment; we referred to their function and one selected published study in each case which provides evidence of the functional involvement of the corresponding gene in PCa. Among these candidates, we found, e.g., *NOTCH3, CDK2AP1, KLK4, ADAM15, HIVEP3, NDUFB7, PPFIA3, MIR4518, ORAI3, CCNE1, CEBPB, REPIN1, DNMT3B, CYTH1,* and *KEAP1.*

## 3. Discussion

At the beginning of our study we focused on screening for differentially hypomethylated whole CpG islands, associated with 5′ regulatory gene regions (“Cor”), and further upstream located CpG shores (“Ups”) (Figure 1 and Figure 2). It has been shown that most methylation alterations in cancer occur not in promoters, nor in CpG islands, but in sequences up to 2 kb distant, which have been termed ‘CpG island shores’. This CpG island shore methylation is related to gene expression and alternative transcription [55].

Our internally validated data reveal many new hypomethylated CpG islands and CpG shores in PCa (Figure 1 and Figure 2). E.g., among the 370 hypomethylated CpG islands and shores found in the PCa samples provided by clinical center No. 2, we found 32 pseudogene loci, e.g., *UBE2CP5*, *RNY5P6*, *OR52P2P*, *OR7E1P,* and *RPS17P7*, and 19 miRNA and 3 lncRNA loci which are hypomethylated. E.g., among the miRNAs we found *MIR-662*, *MIR-524*, and *MIR-30e*, which had been previously shown by others to be deregulated in PCa, and were suggested as diagnostic miRNAs in PCa [56]. Furthermore, *MIR-662* is described to induce chemoresistant and oncogenic phenotypes and to be associated with increased risk of distant metastasis and a poor prognosis in squamous cell carcinoma [57]. It affects the expression of genes which activate EMT, cell adhesion, and WNT pathways, and interestingly, the abundance of transcripts of long non-coding RNAs (lncRNAs) and pseudogenes [57]. WNT pathways are comprehensively described to have a fundamental role in prostate carcinogenesis, metastasis, and therapy resistance, where drugs that specifically target WNT signaling components are thought to reach the clinic soon [58]. *MiRNA-769-5p*, which we also found hypomethylated, has been recently introduced as an oncogene in PCa [59]. Its expression was found inversely associated with patient survival, and its inhibition reduces proliferation and increases apoptosis of PCa cells [59]. We found the lncRNA*LHFPL3-AS1* hypomethylated in PCa, which has been shown to be upregulated in melanoma and to contribute to tumorigenesis of melanoma stem cells by suppressing apoptosis through inhibition of Bcl-2 mRNA degradation [60]. *MAFG1-AS1*, hypomethylated in our data, has been reported to be highly expressed in tumor tissues and cells and to act as a promoter of tumor progression in diverse cancer types, e.g., breast [61] and colorectal cancer [62].

Interestingly, we also found dozens of genes to be associated with a hypomethylated CpG island or “shore” at their 5′ region in PCa, from which we know that normally their expression is confined to a specific cell type in which they exert a highly specialized function. For instance, the following genes can be attributed to those: Complement C1r (*C1R*), Heme Oxygenase 1 (*HMOX1*), Syncollin (*SYCN*), Acrosin-Binding Protein (*ACRBP*), Achaete-Scute Family BHLH Transcription Factor 2 (*ASCL2*), Olfactory Receptor Family 10 Subfamily H Member 4 (*OR10H4*), Complement C3 (*C3*), Protein C Receptor (*PROCR*), HYDINAxonemal Central Pair Apparatus Protein (*HYDIN*), Myosin Heavy Chain 4 (*MYH4*), Fetuin B (*FETUB*), and Killer Cell Immunoglobulin-Like Receptor, two Igdomains and long cytoplasmic tail 3 (*KIR2DL3*). Of note, *HMOX1*, *ACRBP*, *FETUB*, and *KIR2DL3* are overexpressed in PCa [24]. E.g., normally NK cells are the main immune cells expressing *KIR2DL3* and displaying *KIR2DL3* hypomethylated gene promoters, using a unique clonotypic expression mode which has been shown to be governed by differential DNA methylation and chromatin organization of the small CpG island at the 5′-gene region [41,42,63].

On the other hand, we found many hypomethylated genes in our PCa sample cohorts that have been reported to be functionally involved in cancer and PCa development and which, in accordance to their methylation status, have been shown to be overexpressed in PCa [24] (Table 2). For instance, *MENIN 1* has been identified as a co-activator of AR signaling and a potential therapeutic target in advanced PCa. It is upregulated in castration-resistant PCa, and high MENIN expression correlates with poor overall survival [64].

Eph receptor A10 (*EphA10*) gene has been found hypomethylated in PCa by us and Nagano et al., who reported that it is overexpressed in breast cancer and PCa, and its targeting with an anti-EphA10 mAb results in cytotoxicity in EphA10 positive PCa cells [40]. *B4GALNT4*, which we found hypomethylated, has been described to be upregulated in breast, lung, and PCa. In PCa it is involved in beta-N-acetylgalactosaminylation of prostate-specific antigen (PSA) during prostate carcinogenesis, and it is a potential marker of unfavorable prognosis in lymph node-negative locally advanced PCa [26]. *CDK2AP1*, which we found hypomethylated, has been shown to correlate with an enhanced demethylation and expression of the AR gene in PCA, to induce cell cycle arrest and apoptosis, and to reduce the invasive ability of PCa cells [38].

These putative functional-relevant hypomethylations have been found in PCa samples from independent clinical centers No. 1 and No. 2. In the samples from No. 2, few hypomethylations consistently prevail in 10, 9, and 8 out of 10 PCa samples. Therefore, it can be assumed that combinations of those hypomethylations might be useful for the identification of PCa and its clear distinction from benign prostatic hyperplasia tissue material. Of note, a higher number of samples in this stage of the study could provide a more substantial conclusion.

However, in order to take advantage of differential methylation for diagnostic purposes, a high resolution DNA methylation screening analysis is needed [19]. Meanwhile, it is established that beyond the classical epigenetic dogma portraying the function of DNA methylation at CpG islands as an inhibitor of gene expression, it is known that genome-wide distributed DNA methylation at resolution of CpG dinucleotides plays functional roles in chromatin plasticity, gene regulation, and splicing [65,66,67]. Additionally, the carefully scrutinized computational biology analysis of our CpG islands data encouraged us to draw our attention to the inspection of the CpG-rich 60 bp probes, as they have been used in our DNA methylation array analyses, in order to increase resolution.

Indeed, on that basis of short CpG-rich fragments, we detected many DMRs which consistently prevail in all 20 PCa samples from clinical center No. 3, but not in the reference samples. Hence, this approach provides an enhanced resolution of hypomethylation detection. In our strategy to develop reliable biomarkers, we will go forward to analyze these DMRs by bisulfite genomic sequencing to reveal the exact positions of the differentially methylated CpG dinucleotides. Based on this, we will be able to define the most suitable, discriminating primer-binding regions in newly designed MSPCR assays for diagnosis, as we have previously described it [68]. As reported in detail, this method allows, by applying idiolocal normalization of real-time methylation-specific PCR [69], a fast, cost-effective, and reliable detection of a given PCa specific differential methylation, even in cancer samples which harbor heterogenic genetic material.

Furthermore, among the 223 CpG-enriched differential methylated probes coming up from the comparison of these PCa samples with the PCa adjacent samples (clinical center No. 3), we found the following genes, three pseudogenes, *EIF4A2P5*, *RNU6-627P*, and *RN7SL671P*, two *MiRNAs*, *MIR4285* and *MIR3176*, several lncRNAs, e.g., *PTGES2-AS1*, *IBA57-AS1*, *SCAMP1-AS1*, *OGFR-AS1*, *CREB2-AS1*, *TTC39A-AS1*, and *ST3GAL6-AS1*, to be associated with many cell type specific expressed and functioning genes, as e.g., Espin-Like, required for the formation and maintenance of inner ear hair cell stereocilia and staircase formation [70]. Dynein Axonemal Intermediate Chain 1, the encoded protein, is part of the dynein complex in respiratory cilia [71]. Synaptotagmin 17 controls neurite outgrowth and synaptic physiology [72]. Craniofacial Development Protein 1 is involved in embryogenesis [73] and the maintenance of higher-order chromatin organization [74]. Recently, a deregulated *MIR3176* has been discovered in the exosomes of chemoresistant PCa cells, and it has been suggested that mainly androgen receptor (*AR*) and phosphatase and tensin homolog (*PTEN*) target genes are mainly influenced by this [75]. *MIR4285*, which overlaps with a CpG island [76], has been described to be deregulated in Serum MicroRNA profiles of patients with colon cancer [77]. Recently, *TTC39A-AS1* has been revealed to be a critical regulator in the tumorigenicity of patients with breast cancer, where a high level of *TTC39A-AS1* correlates with a shorter overall survival. Functionally, the absence of *TTC39A-AS1* accelerates cell apoptosis, but retains cell proliferation, migration, and invasion [78]. Additionally, also, recent results suggested that upregulated lncRNA *ST3GAL6-AS1* promotes adhesion and invasion of multiple melanoma cells [79].

Finally, we also identified hypomethylated CpG-rich probes which consistently prevail in all 20 tumor samples from clinical center No. 3. They are associated with overexpression according to The Cancer Genome Atlas (TCGA) PCa expression data bank. Additionally, the literature provided evidence of their crucial role in PCa and cancer (Table 2). It is foreseeable to combine several of these promising markers to further investigate their potential for clearly discriminating PCa in, e.g., mixed tissues of questionable biopsies. Following this strategy, we would choose, for instance, the DMRs associated with *NOTCH3*, whose overexpression has been significantly associated with lymph node metastasis, higher pT stages, higher pathological tumor stages, and groups of higher grades, reflecting features of aggressive tumors in PCa [80,81]. These would be analyzed together with DMRs of *ADAM15*, which has been reported to be strongly upregulated in a highly aggressive fraction of PCa [47] and to support PCa metastasis [82]. These exemplary PCa hypomethylations could be complemented by *DNMT3B*, which has been shown to be highly abundant in PCa cells and to regulate tumorigenicity [52]. Targeting DNMT3B induces a resensitization to Enzalutamide, an FDA approved AR antagonist for the treatment of PCa patients [83]. A further candidate would be *KLK4*, which has been implicated in PCa [46]. Again, first we have to dissect the exact differential methylated CpG positions associated within these genes in order to design the best discriminating primers for MSPCR. Then, we would apply this and other marker combinations in order to determine which combinations provide the highest sensitivities and specificities from positive and negative biopsy samples. We share the view that global PCa specific hypomethylation may occur as a direct consequence of grave methylgroup-metabolism disturbances [25,26], and we observed that these occur in many loci which may functionally be not relevant for PCa. The PCa cells tolerate these abnormal methylation changes and the consequences for the altered transcriptional competence of these epigenetically affected loci. We named these pleiotropic DMRs, and seemingly they are the majority. On the other hand, functional relevant hypomethylations occur also, which result in gene expression differences of loci with functional relevance for PCa. Here, we think that differential methylation at distinct CpG dinucleotides may exert an influence directly or indirectly, e.g., by influencing lncRNA and miRNA expression, to contribute to a PCa specific genome usage. Our data provide evidence that we are able to dissect short, differential methylated CpG-rich DNA fragments and combinations of them which are consistently present in all tumors. We would call them tumor cell-specific differential methylated CpG dinucleotide signatures (TUMS). This is in accordance with the recent demonstration that individual CpGs are consistently hypomethylated in specific cell types, and can be used to estimate the fraction of a specific cell type or the composition of cellular mixes and tissues [9,84]. Our notion is that a tumor cell of a certain developmental stage with distinct features should harbor in between the huge amount of pleiotropic and heterogeneous, differential methylated CpGs, showing a characteristic and consistent, differential methylated CpG signature, associated with distinct features, which could serve as an epigenetic source for diagnosis, prognosis, and follow up. Thus, an aim is to introduce such TUMS into clinical practice to support initial diagnosis by the pathologist, and later on even to provide new TUMS, which provide a prognostic stratification of PCa. A specific TUMS to predict recurrence would solve a crucial challenge in today’s urology.

Hence, a further major question which arises from our point of view and which we will follow is whether an aggressive PCa which will metastasize and kill the patient harbors characteristic hypomethylated CpG positions, which reflect the feature of aggressiveness and are not present within the genome of an indolent PCa. In our next study we therefore will compare indolent PCa samples from active surveillance patients with the few from those who had to leave active surveillance because their tumor suddenly became aggressive and metastasized, in order to computationally dissect TUMS characteristic for this aggressiveness. This strategy has been suggested by our esteemed clinical advisor. The identification of these aggressive PCa DMR signatures will give us a new tool to stratify PCa from biopsies. The basic assumption behind this new approach is that any cancer cell type and its clinical relevant feature share a corresponding specific TUMS reflecting this phenotypic feature and which is of highest value for prognosis. On that basis this platform technology will serve for the development of diagnostic and prognostic epigenetic biomarkers for any cancer entity, and furthermore, it reveals new functional relevant single CpG positions as potential targets for therapy.

Our hierarchical clustering analyses in Figure 5 reveals that a classification of PCa samples in distinct groups is feasible based on methylated CpG-rich probes of 60 nucleotides. The correlation with distinct clinical features of these subgroups could lead to a useful new epigenetic tumor classification system of PCa, especially when we will be able to confine this analysis to the functional relevant hypomethylations, discarding all pleiotropic and inconsistently occurring hypomethylations. Finally, in accordance with the existent literature, Panther analyses applied on our data reveal, among others, angiogenesis, NOTCH, Toll receptor, WNT, and p53 signaling pathways as main pathways involved in PCa, suggesting that distinct, until now cryptic hypomethylation events underlie those well-established PCa associated pathway alterations (Figure 6).

## 4. Materials and Methods

### 4.1. Cell Line and Tissue Samples

The cell LNCaP (Lymph Node Carcinoma cells of the Prostate) was cultured in RPMI-1640 (Gibco Life Technologies, Karlsruhe, Germany), supplemented with 10% fetal calf serum and 100 mg/mL penicillin/streptomycin. DNA from the cell line PrEC (Human Prostate Epithelial Cells) was provided by Dr. Michele Hoffman, Department of Urology, Medical Faculty, Heinrich Heine University Düsseldorf. All methods and data were used in accordance with relevant guidelines and regulations. Ethical Approval was granted by the Erasmus MC Medical Ethics Committee according to the Medical Research Involving Human Subjects Act (MEC-2004-261; MEC-2010-176). Additionally, ethical approval was granted, Studien-Nr.: 2022-1982, by the Ethics Committee of the medical faculty of the Heinrich-Heine-Universität Düsseldorf.

The following prostate samples were used from three different European clinical centers:

1. No. 1: University Hospital of Düsseldorf (UKD), *Tumorbank*

Histological H&E stained sections from formalin-fixed paraffin-embedded (FFPE) tissue specimens from prostatectomies and biopsies. Five samples of Gleason 4 + 3 (prostatectomies) and five samples of Gleason 3 + 4 (Biopsies). They were pathologically reviewed for tumor (>90%), tumor/adjacent healthy tissue (50/50%), BPH (>90%) content, and the targeted area was marked by a trained pathologist (Dr. Braunstein, Prof. Anlauf, pathology Düsseldorf), cut into 5 μm slices, microdissected and transferred into Eppendorf reaction tubes. Briefly, FFPE samples were deparaffinized with xylene, washed twice with ethanol, dried 10 min at 37 °C and resuspended in 200 μL incubation buffer containing 2 mg/mL proteinase K. Samples were incubated overnight at 70 °C and mixed with 400 μllysis buffer. Lysates from FFPE tissue were transferred to well 1 of the supplied cartridge of the corresponding kit, and DNA was automatically purified and eluted in 30 μL Tris-buffer, pH 8.0 by the Maxwell instrument. The yield ranged between 1 and 8 μg of high quality DNA per sample. Purity control and quantification were performed using a NANODROP 2000 UV–Vis spectrometer (Thermo SCIENTIFIC, Wilmington, NC, USA). DNA from FFPE samples was isolated using the Maxwell 16 FFPE Tissue LEV DNA Purification Kit (Promega, Madison, WI, USA, #AS1130) according to the manufacturer’s recommendations. All methods were carried out in accordance with relevant guidelines and regulations. We confirm that the experimental protocols were approved and informed consent was obtained from all participants.

2. No. 2: University Hospital, Clinic of Urology, Tübingen

10 PCa tissue samples (prostatectomies) of pT2c G3 + 4, pT2a G3 + 4, pT3b G4 + 3, pT2a G3 + 4, pT2c G4 + 3, pT2b G3 + 3, pT2c G4 + 5, pT2c G3 + 4, pT2c G3 + 4, pT2c G3 + 4 were prepared as described above in 1. to obtain DNA from nearly >95% of tumor cells. PCa staging and grading were performed by a trained genitourinary pathologist.

3. No. 3: Erasmus Medical Center Rotterdam

20 DNA samples provided from tumor tissues consisting of 80–100% cancer cells. pT4b G3 + 4, pT3a G3 + 4, pT2c G3 + 3, pT2a G3 + 4, pT2c G3 + 3, pT3a G3 + 3, pT2b G 3 + 3, pT3a G3 + 3, pT4a G 3 + 3, pT4x G3 + 3, pT3c G3 + 3, pT3x G3 + 3, pT2c G3 + 4, pT4a G3 + 4, pT2a G 3 + 3, pT2c G 3 + 4, pT2x G 3 + 3, pT2c G3 + 4, pT3c G 3 + 4, pT4a G3 + 3. A total of 8 DNA samples provided from tumor tissue adjacent healthy prostate gland tissue (95%). PCa staging and grading were performed by a trained genitourinary pathologist.

### 4.2. Methylated DNA Immunoprecipitation (MeDIP)

MeDIP has been performed in the past, as we have previously described it in detail (2018). In brief, 1 μg of genomic DNA dissolved in a final volume of 100 μL was sonicated at 4 °C in TPX^®^ polymethylpentene tubes using a Bioruptor^®^ sonicator (Diagenode, Liege, Belgium) to produce random-sized fragments ranging from 300 to 1.000 bp. We used the magMeDIP kit (Diagenode) and the 5-methylcytosine (5-mC) monoclonal 33D3 antibody (Diagenode) for immunoprecipitation.

### 4.3. Amplification and Labelling of DNA

The Genome Plex Complete WGA Kit (Sigma-Aldrich, St. Louis, MO, USA) was used to perform whole genome amplification of the input DNA and the immunoprecipitated DNA samples as described by the user’s guide. The amplification products were purified using QIAquick PCR Purification Kit (Qiagen, Hilden, Germany). Input and IP DNA were labeled with Cyanine 3 and Cyanine 5, respectively, using random primers and Klenow fragment polymerase. For labeling, the SureTagComplete DNA Labeling Kit (Santa Clara, CA, USA) was used, as suggested by the manufacturer. Labeled DNA was cleaned with 70% ethanol and dried using a vacuum centrifuge for few minutes.

### 4.4. Hybridization of Microarrays

Equal amounts of labeled IP and input DNA (700 ng) were combined and loaded on NimbleGen 385 K RefSeq Promoter Arrays HG18, containing all known human RefSeq genes (Roche, Basel, Switzerland), or Agilent Human DNA Methylation 2.1 M Deluxe Promoter Arrays. Initially, samples from Düsseldorf were analyzed by NimbleGen 385 K Arrays; after the upgraded, high resolution 2.1 M Agilent Arrays became available, all other tissue samples were analyzed on these arrays.

On both arrays, all known gene 5′-regulatory regions are covered by 60–75 mer probes with approximately 100-bp spacing. The 385 K arrays cover a region between 2.2 kb downstream and 0.5 kb upstream of the transcription start site (TSS). The 2.1 M arrays cover a region between 7.25 kb downstream and 3.25 bp upstream of the TSS. The hybridization procedure was executed at 42 °C for 16 h in the NimbleGen hybridization chamber in accordance with the manufacturer’s protocol. The hybridized arrays were washed thoroughly and dried using a microarray centrifuge for 1 min in the dark.

### 4.5. DNA Methylation Microarray Scanning and Data Analyses

The hybridized arrays were scanned on an MS200 microarray scanner (Roche, Basel, Switzerland) at a resolution of 2 μm. The raw methylation data were extracted with the default ChIP protocol from software NimbleScan for 385 K arrays and with DEVA for 2.1 M arrays. Methylation ratios between the IP DNA samples and the control input samples were normalized across samples using the quantile method after performing a variance stabilization using log_2_ scaling for each promoter feature on the array. As previously reported [85], we implemented a “democratic” method to select the DMRs more common across patients by counting the “votes” of differences between tumor and control samples. All data processing, including mapping of microarray probes to promoters, gene annotation, data post-processing, hierarchical clustering, identification of DMRs, and graphics, were performed with in-house developed functions in Matlab [86,87]. The promoter loci information on the DNA methylation microarray probes was taken from NimbleGen annotation information based on RefSeq version hg18 and the Agilent on version hg19.

### 4.6. Bisulfite Genomic Sequencing

Bisulfite sequencing was performed following bisulfite conversion with the EpiTec Kit (Qiagen, Hilden, Germany) as described [27,68]. Primers for *KIR2DL3* amplification were applied as described [26,27].

### 4.7. Panther Analysis

We used the PANTHER classification system (http://www.pantherdb.org, accessed on 4 May 2022), which is a comprehensive system combining gene function classifications, pathways, and further tools to enable biologists to analyze genome-wide experimental data. It covers 131 complete genomes organized into gene families and subfamilies [43,44].

## Figures and Tables

**Table 1:** Genes found hypomethylated in three different patient cohorts. Selected genes which were found hypomethylated in all three heat map analyses of hypomethylation are presented in Figure 1 and Figure 2. They were selected by consistency of occurrence in these three patient sample cohorts from two different clinical centers (No. 1 and No. 2) and the existing evidence in the literature for their involvement in PCa and cancer in general. The number of votes, calculated by the democratic method, is the number of hypomethylated tumors from 10 tumor samples compared to the average methylation in 5 BPH samples. The gene expression in PCa according to The Cancer Genome Atlas (TCGA) PCa expression data bank [23,24] is also shown.

Gene Symbol/ Name/ EntrezID/ (Chromosomal Location)	Function	Selected Publication on Gene’s Involvement in PCa and Cancer	Votes in 10 Tumors Compared to BPH	PCa Gene Expression
WBP11-Cor WW domain-binding protein 11 51,729 (12p12.3)	Nuclear protein. Binds to the Npw38WW domain activating pre-mRNA splicing	WBP11 is required for splicing the TUBGCP6 pre-mRNA to promote centriole duplication [25]	9	1.07
B4GALNT4-Ups ß-1,4-N-Acetyl-Galactosaminyltransferase 4 338,707 (11p15.5)	Transfers N-acetylgalactosamine (GalNAc) from UDP-GalNAc to N-acetylglucosamine-ß -benzyl	Differentially expressed genes associated with prognosis in locally advanced lymph node-negative PCa [26]	8	3.66
KIR2DL3-Cor Killer Cell Immunoglobulin-Like Receptor, Two Ig Domains And Long Cytoplasmic Tail 3 3804 (19q13.42)	Inhibits activity of NK cells, preventing cell lysis	KIR 2D (L1, L3, L4, S4) and KIR 3DL1 protein expression in non-small cell lung cancer [27]	10	1.38
TAF6L-Cor TATA-Box-Binding Protein Associated Factor 6-Like 10,629 (11q12.3)	Member of the PCAF complex, an epigenetic regulator essential for somatic reprogramming	TAF5L and TAF6L maintain self-renewal of embryonic stem cells via the MYC regulatory Network [28]	10	1.36
MEN1-Ups Menin 1 4221 (11q13.1)	Tumor suppressor functions in histone modification and epigenetic gene regulation by altering chromatin structure	Menin enhances androgen receptor-independent proliferation and migration of PCa cells [29]	10	1.33
HMOX1-Cor Heme Oxygenase 1 3162 (22q12.3)	HO catalyzes the degradation of heme. Has an anti-apoptotic function	PTEN deletion and heme oxygenase-1 overexpression cooperate in PCa progression and are associated with adverse clinical outcome [30]	10	1.06
WBSCR17-Cor Polypeptide N-Acetylgalactosaminyltransferase 17 64,409 (7q11.22)	Predicted to play a role in membrane trafficking	The identification of specific methylation patterns across different cancers [31]	10	1.07
NDUFB7-Cor NADH:Ubiquinone Oxidoreductase Subunit B7 4713 (19p13.12)	Subunit of NADH dehydrogenase (Complex I). Functions in the transfer of electrons from NADH to the respiratory chain	Cholesterol uptake and regulation in high-grade and lethal PCas [32]	9	1.11
BRD4-Cor Bromodomain-Containing 4 23,476 (19p13.12)	Chromatin reader. Recognizes and binds acetylated histones. Plays a key role in transmission of epigenetic memory across cell divisions and transcription regulation	BRD4 regulates key transcription factors that drive epithelial-mesenchymal transition in castration-resistant PCa [33]	8	0.997
PPFIA3-Cor PTPRF-Interacting Protein Alpha 3 8541 (19q13.33)	Localize receptor-like tyrosine phosphatases type 2A at specific sites on the plasma membrane	Establishment of a DNA methylation marker to evaluate cancer cell fraction in gastric cancer [34]	8	1.35
MIR4518-Cor microRNA 4518 100616405 (16p11.2)	Post-transcriptional regulation of gene expression	LncRNA SNHG16 functions as an oncogene by sponging MiR-4518 and up-regulating PRMT5 expression in glioma [35]	10	ND
EPH10 EPH Receptor A10 284,656 (1p34.3)	Receptor tyrosine kinases (RTKs), and their ephrin ligands, such as EPH10, are important mediators of cell–cell communication regulating cell attachment, shape, and mobility in neuronal and epithelial cells	Eph receptor A10 has a potential as a target for a PCa therapy [36]	10	5.28
ORAI3 ORAI Calcium Release-Activated Calcium Modulator 3 93,129 (16p11.2)	Ca^(2+)^ release-activated Ca^(2+)^-like (CRAC-like) channel subunit which mediates Ca^(2+)^ influx and increase in Ca^(2+)^-selective current by synergy with the Ca^(2+)^ sensor, STIM1	Overexpression of certain transient receptor potential and Orai channels in PCa is associated with decreased risk of systemic recurrence after radical prostatectomy [37]	10	1.07
CDK2AP1 Cyclin-Dependent Kinase 2 Associated Protein 1 8099 (12q24.31)	Specific inhibitor of the cell-cycle kinase CDK2. It plays a role in cell-cycle and epigenetic regulation	Cell cycle regulator cdk2ap1 inhibits PCa cell growth and modifies androgen-responsive pathway function [38]	9	1.04

**Table 2:** Gene-associated CpG-rich probes hypomethylated in PCa. This table presents selected genes which were found associated with hypomethylated CpG-enriched probes in the heat map analyses of hypomethylation presented in Figure 4, right panel. They were selected by consistency of occurrence in all cancer patient samples of the No. 3 cohort and the existing evidence in the literature for their involvement in PCa and cancer in general. The number of votes, calculated by the democratic method, is the number of hypomethylated tumors from 20 tumor samples compared to the average methylation in 8 tumor-adjacent healthy samples. The gene expression in PCa according to The Cancer Genome Atlas (TCGA) PCa expression data bank [23,24] is also shown.

Gene Symbol/ Name/ Entrez ID/ (Chromosomal Location)	Function	Selected Publication on Gene’s Involvement in PCa and Cancer	Votes in 20 Tumors Compared to Tumor-Adjacent Healthy Samples	PCa Gene Expression
*NOTCH3* Notch Receptor 3 4854 (19p13.12)	Receptor for membrane-bound ligands Jagged1, Jagged2, and Delta1 to regulate cell-fate determination. Affects differentiation, proliferation, and apoptotic programs	Notch signaling dynamics in the adult healthy prostate and in prostatic tumor development [45]	20	1.11
*CDK2AP1* Cyclin-Dependent Kinase 2 Associated Protein 1 8099 (12q24.31)	Forms a core subunit of the nucleosome remodeling and histone deacetylation (NURD) complex that epigenetically regulates embryonic stem cell differentiation. Specific inhibitor of the cell-cycle kinase CDK2	Cell cycle regulator Cdk2ap1 inhibits PCa cell growth and modifies androgen-responsive pathway function [38]	20	1.04
*KLK4* Kallikrein-Related Peptidase 4 9622 (19q13.41)	Serine protease with major role in enamel formation	KLK4 induces anti-tumor effects in human xenograft mouse models of orthotopic and metastatic PCa [46]	20	1.86
*ADAM15* ADAM Metallopeptidase Domain 15 8751 (1q21.3)	Metalloproteinase with gelatinolytic and collagenolytic activity. Inhibits ß-1 integrin-mediated cell adhesion and migration of airway smooth muscle cells. Cleaves E-cadherin in response to growth factor deprivation	Overexpression of the A disintegrin and metalloproteinase ADAM15 is linked to a small but highly aggressive subset of PCas [47]	20	1.32
*HIVEP3* Human Immunodeficiency Virus Type I Enhancer-Binding Protein 3 59,269 (1p34.2)	TF that regulates nuclear factor κB-mediated transcription by binding the κBmotif in target genes. Strongly inhibits TNF-α-induced NF-κB activation. Interaction with TRAF proteins inhibits both NF-κB-mediated and c-Jun N-terminal kinase/JNK-mediated responses that include apoptosis and proinflammatory cytokine gene expression	Combined overexpression of HIVEP3 and SOX9 predicts unfavorable biochemical recurrence-free survival in patients with PCa [48]	20	1.17
*NDUFB7-Cor* NADH:Ubiquinone Oxidoreductase Subunit B7 4713 (19p13.12)	Subunit of NADH dehydrogenase (Complex I) that functions in the transfer of electrons from NADH to the respiratory chain	Cholesterol uptake and regulation in high-grade and lethal PCas [32]	20	1,11
*PPFIA3-Cor* PTPRF-Interacting Protein Alpha 3 8541 (19q13.33)	Localize receptor-like tyrosine phosphatases type 2A at specific sites on the plasma membrane, possibly regulating their interaction with the extracellular environment and their association with substrates	Establishment of a DNA methylation marker to evaluate cancer cell fraction in gastric cancer [34]	20	1.35
*MIR4518-Cor* microRNA 4518 100616405 (16p11.2)	Post-transcriptional regulation of gene expression	LncRNA SNHG16 functions as an oncogene by sponging MiR-4518 and up-regulating PRMT5 expression in glioma [35]	20	ND
*ORAI3* ORAI Calcium Release-Activated Calcium Modulator 3 93,129 (16p11.2)	Ca^(2+)^ release-activated Ca^(2+)^-like (CRAC-like) channel subunit which mediates Ca^(2+)^ influx and increase in Ca^(2+)^-selective current by synergy with the Ca^(2+)^ sensor, STIM1	Overexpression of certain transient receptor potential and Orai channels in PCa is associated with decreased risk of systemic recurrence after radical prostatectomy [37]	20	1.07
*CCNE1* Cyclin E1 898 (19q12)	Essential for the control of the cell cycle at the G1/S (start) transition	PKMYT1 is associated with PCa malignancy and may serve as a therapeutic target [49]	20	1.02
*CEBPB* CCAAT Enhancer-Binding Protein Beta 1051 (20q13.13)	TF regulating expression of genes involved in immune and inflammatory responses. Promotes osteoblast differentiation and osteoclastogenesis	CCAAT enhancer-binding protein beta promotes tumor growth and inhibits apoptosis in PCa by methylating estrogen receptor beta [50]	20	0.612
*REPIN1* Replication Initiator 1 29,803 (7q36.1)	Sequence-specific double-stranded DNA-binding protein required for initiation of chromosomal DNA replication	AP4 modulated by the PI3K/AKT pathway promotes PCa proliferation and metastasis of prostate cancer via upregulating L-plastin [51]	20	1.48
*DNMT3B* DNA Methyltransferase 3 Beta 1789 (20q11.21)	Required for genome-wide de novo methylation. Essential for the establishment of DNA methylation patterns during development	DNMT1 and DNMT3B regulate tumorigenicity of human PCa cells by controlling RAD9 expression through targeted methylation [52]	20	1.37
*CYTH1* Cytohesin 1 9267 (17q25.3)	Promotes activation of ARF factors by replacement of GDP with GTP. Plays an important role in membrane trafficking during junctional remodeling and epithelial polarization	Inhibition of cytohesin-1 by siRNA leads to reduced IGFR signaling in PCa [53]	20	1.1
*KEAP1* Kelch-Like ECH Associated Protein 1 9817 (19p13.2)	Substrate-specific adapter of a BCR E3 ubiquitin ligase complex. Regulates response to oxidative stress by targeting NFE2L2/NRF2 for ubiquitination	Regulation of canonical oncogenic signaling pathways in cancer via DNA methylation [54]	20	1.11

## Data Availability

The data presented in this study are available on request due to privacy/ethical restrictions.

## Supplementary Materials

The following supporting information can be downloaded at: https://www.mdpi.com/article/10.3390/ijms24010386/s1, Figure S1: Hierarchical clustering of all samples used for the internal validation, performed by applying the correlation metric and the average linkage method. Sample 8 was misstated as a BPH control.
