# Supplementary material for: Consistent DNA Hypomethylations in Prostate Cancer
Source: Int J Mol Sci. 2022 Dec 26;24(1):386. doi: 10.3390/ijms24010386 (PMC9820221; doi:10.3390/ijms24010386)
Supplement: Supplementary file 1 [file ijms-24-00386-s001.zip › ijms-2050629-supplementary.pdf]

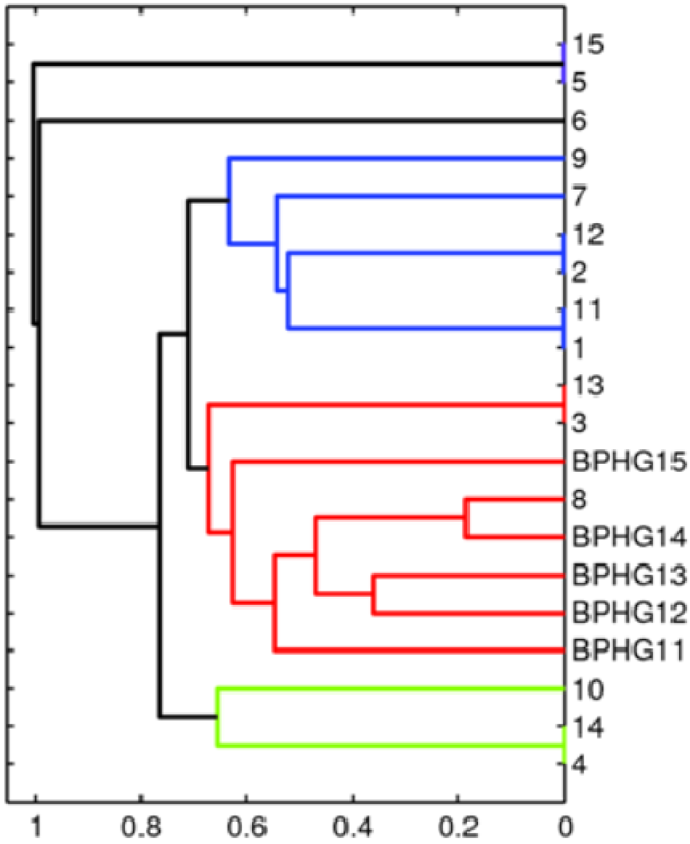

Figure S1 Hierarchical clustering of all samples used for the internal validation, performed by applying the correlation metric and the average linkage method. Sample 8 was misstated as a BPH control.
